# Black goes green: single-step solvent exchange for sol-gel synthesis of carbon spherogels as high-performance supercapacitor electrodes†

**DOI:** 10.1039/d3ya00480e

**Published:** 2024-01-08

**Authors:** Miralem Salihovic, Emmanuel Pameté, Stefanie Arnold, Irena Sulejmani, Theresa Bartschmid, Nicola Hüsing, Gerhard Fritz-Popovski, Chaochao Dun, Jeffrey J. Urban, Volker Presser, Michael S. Elsaesser

**Affiliations:** a Chemistry and Physics of Materials, University of Salzburg 5020 Salzburg Austria michael.elsaesser@sbg.ac.at; b INM – Leibniz Institute for New Materials, Campus D2 2 66123 Saarbrücken Germany volker.presser@leibniz-inm.de; c Department of Materials Science & Engineering, Saarland University, Campus D2 2 66123 Saarbrücken Germany; d Institute of Physics, Montanuniversitaet Leoben 8700 Leoben Austria; e The Molecular Foundry, Lawrence Berkeley National Laboratory Berkeley Berkeley CA 94720 USA; f Saarene – Saarland Center for Energy Materials and Sustainability, Campus C4 2 66123 Saarbrücken Germany

## Abstract

Nanoporous carbon materials with customized structural features enable sustainable and electrochemical applications through improved performance and efficiency. Carbon spherogels (highly porous carbon aerogel materials consisting of an assembly of hollow carbon nanosphere units with uniform diameters) are desirable candidates as they combine exceptional electrical conductivity, bespoke shell porosity, tunability of the shell thickness, and a high surface area. Herein, we introduce a novel and more environmentally friendly sol-gel synthesis of resorcinol-formaldehyde (RF) templated by polystyrene spheres, forming carbon spherogels in an organic solvent. By tailoring the molar ratio of resorcinol to isopropyl alcohol (R/IPA) and the concentration of polystyrene, the appropriate synthesis conditions were identified to produce carbon spherogels with adjustable wall thicknesses. A single-step solvent exchange process from deionized water to isopropyl alcohol reduces surface tension within the porous gel network, making this approach significantly time and cost-effective. The lower surface tension of IPA enables solvent extraction under ambient conditions, allowing for direct carbonization of RF gels while maintaining a specific surface area loss of less than 20% compared to supercritically dried counterparts. The specific surface areas obtained after physical activation with carbon dioxide are 2300–3600 m^2^ g^−1^. Transmission and scanning electron microscopy verify the uniform, hollow carbon sphere network morphology. Specifically, those carbon spherogels are high-performing electrodes for energy storage in a supercapacitor setup featuring a specific capacitance of up to 204 F g^−1^ at 200 mA g^−1^ using 1 M potassium hydroxide (KOH) solution as the electrolyte.

## Abstract 2

The novel synthesis of carbon spherogels in organic solvents enables energy and solvent savings and omits supercritical drying. In addition, the resulting carbon spherogels feature excellent capacitance for energy storage in a supercapacitor setup.

## Graphical abstract

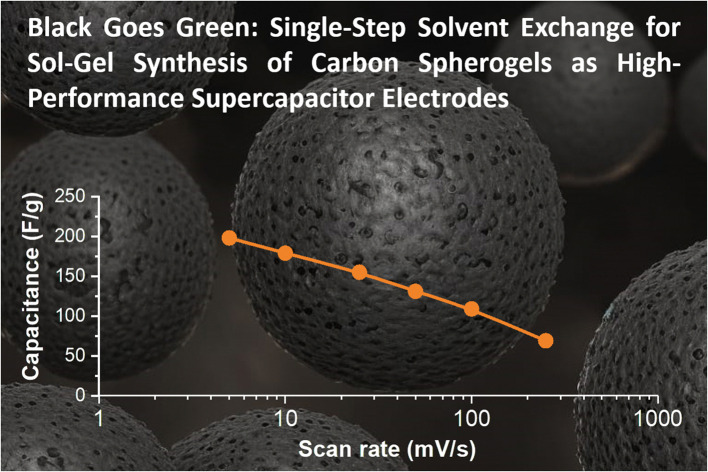

## Introduction

Research and development of advanced, high-performance nanoporous carbons have attracted significant interest for various applications such as energy storage,^1^ catalysis,^2^ medicine,^3^ or separation processes.^4^ Regarding the synthetic pathways, further actual aspects concentrate on sustainable precursors,^5,6^ materials-savings and energy-savings during production and possible recyclable usage during applications. Carbon spherogels are relatively new carbon aerogel variants that we first introduced in 2019.^7^ The unique feature of carbon spherogels is a network morphology, which solely consists of hollow carbon spheres. Each hollow sphere is tailorable concerning its inner diameter and wall thickness and displays high geometrical uniformity concerning the bulk network. This feature ensures an exceptional homogeneity and reproducibility of material properties. Furthermore, carbon spherogels retain porosity features of analogous, non-templated carbon aerogels with the same bulk density and sol composition.^7^ As a result, a unique material property combination of carbon aerogel and hollow carbon sphere structures for energy storage applications is achieved. The hollow carbon sphere structure yields additional benefits, for example, mechanical stability during charge/discharge cycles or improved electrolyte wetting.^8^ We demonstrated the direct use of binder-free carbon spherogel slices as electrode materials in supercapacitors with a coin-cell setup by first electrochemical investigations. In contrast to classical, non-templated, and pearl-bead-like carbon aerogel structures, carbon spherogel structures withstand at least 10 000 charge/discharge cycles with no visible damage and capacitance retention above 95%.^9^

Hybrid hollow carbon sphere materials have been the focus of recent investigations due to improved performance in energy storage applications. Introducing guest metal species may lead to additional tailorable functionality in batteries, which improves electrolyte diffusion kinetics or may catalyze electrochemical reactions (for instance, oxygen reduction reaction (ORR), oxygen evolution reaction (OER), or hydrogen evolution reaction (HER)) in fuel cells.^10^ As a first example, hybrid carbon spherogels were prepared by functionalizing resorcinol in a Ti(acac)_2_(OiPr)_2_ solution, which yielded a distinct titania layer inside the microporous carbon wall.^11^

In the past, carbon aerogels have been prepared mainly by the pyrolysis of a resorcinol–formaldehyde aerogel formed by polycondensation reactions in aqueous media.^12^ When using water, controlling the pH *via* catalysts allows for designing the aerogel nanostructure.^13^ Carbon aerogels of various densities have previously been prepared *via* gelation and drying in isopropyl alcohol, which has been described to be faster and easier than conventional processes, using hydrochloric acid and hexamethylene triamine (HMTA) as catalysts.^14,15^ One of the main challenges in aerogel production is removing the liquid component of a gel without material damage due to capillary strains and loss of porosity and specific surface area.^16^ Various strategies have been investigated to strengthen the resorcinol–formaldehyde gel network and facilitate drying with low material shrinkage. Past works include condensation of hydroxymethyl groups by treatment with trifluoroacetic acid,^17^ incorporation of graphene oxide into the RF network as an anti-shrinkage additive,^18^ or additional crosslinking by isocyanates.^19^

In the present study, we introduce a novel preparation of carbon spherogels *via* resorcinol–formaldehyde gelation, catalyzed by trimethylamine, in an organic solvent, namely IPA. Compared to previous water-based studies, several advantages regarding simplicity, energy- time- and carbon dioxide emission savings evoke: First, by using an organic solvent, (1) the solvent exchange of commonly used water is avoided before supercritical drying, (2) the surface tension of the solvent is reduced in comparison to water, facilitating ambient drying, and (3) an opportunity for the future use case of metal oxide incorporation and preparation of hybrid variants is given due to the solubility of metal oxides in IPA. Furthermore, we demonstrate the ability of these materials to be directly carbonized without an additional solvent extraction step and significant loss of specific surface area (SSA), retaining at least 80% of the value of supercritically dried analogs. The directly carbonized IPA-carbon spherogel variants enable faster and cheaper production with conventional laboratory equipment. The benefits of hollow carbon sphere (HCS) materials (increased rate capability and capacitance) can be optimized regarding the required time and cost, making this synthesis route attractive for industrial applications. Thick-walled and thin-walled carbon spherogel materials were investigated as electrode materials in supercapacitors. In addition, this synthesis route opens the door to a facile production of hybrid HCS variants for future investigations.

## Experimental

### Materials and synthesis

Polystyrene nanospheres (PS, 250 nm diameter size, polydispersity index (PDI) < 0.1) were prepared in an emulsifier-free emulsion polymerization process,^20^ using polyvinylpyrrolidone (PVP; average molar weight 40,000) as the stabilizer and potassium persulfate (>99.0% purity) as the initiator. Styrene (≥99% purity) was used without further purification. After polymerization, the aqueous PS solution was centrifuged overnight, and the separated PS nanospheres were transferred into a technical-grade solution of IPA (technical). The product was centrifuged and washed with replenished IPA three times. The final product was stored as the stock solution, and the PS amount (in mass%) was determined by thermogravimetric analysis (TGA).

Resorcinol (R; 99% purity) was first dissolved in a PS/IPA solution (in a molar ratio of 0.016 to 0.128). In a typical synthesis, 1.84 g of R was dissolved in 15.72 g of a PS/IPA solution containing 2 mass% PS (R/IPA 0.064). After five minutes at low-rate stirring, which was kept constant throughout, 2.72 g formaldehyde (F; aqueous, 37%, stabilized with 10% methanol) was added. Five minutes later, 1.67 mL trimethylamine (TEA; 1 M in IPA); molar ratio of (R/TEA = 10) was added dropwise, and the sol was stirred for 60 min. Finally, the sol was transferred to glass molds (inner diameter 12.5 mm) and placed in a drying cabinet for gelation over 7 days at 80 °C. Afterward, the samples were washed once with technical grade IPA. One part of the batch was directly carbonized (DC) from its organogel state. The other part of the batch was supercritically dried (SCD) with carbon dioxide at 110 bar and 60 °C, followed by carbonization. All carbonization steps were performed in a tube furnace under an argon atmosphere. The samples were heated to 60 °C with a dwell time of 4 h and then to 800 °C with a dwell time of 2 h under constant argon flow (75 NL h^−1^; NL: standard liter).

Subsequently, the samples were physically activated by heating to 900 °C under constant argon flow and dwelling for 90 min under constant CO_2_ flow (1 NL h^−1^), then cooling to room temperature under continuous argon flow.

### Materials characterization

Thermogravimetric analysis was performed on a NETZSCH STA 449 F3 Jupiter device from 20 °C to 1000 °C (argon atmosphere). Dynamic light scattering measurements were taken on a Malvern Zetasizer instrument using a light-backscattering angle of 173°. One measurement consisted of 3 × 30 separate sub-measurements.

Carbon spherogel was further analyzed with a JEOL JEM F200 transmission electron microscope (TEM) using an accelerating voltage of 20 kV. The instrument was equipped with a cold-field emission source and used a TVIPS F216 2k by 2k CMOS camera. For sample preparation, the powder of the samples was placed on a TEM grid consisting of a lacey carbon film deposited on a Cu grid. Micrographs of carbon spherogels were recorded on a Zeiss Ultra Plus field emission scanning electron microscope (SEM) by an in-lens detector. The acceleration voltage was adjusted to 4 kV.

For nitrogen adsorption, a Micromeritics ASAP 2420 sorption system was used at −196.15 °C. The samples were degassed at 300 °C for 24 h before the measurements. Specific surface areas and pore size distributions were calculated by two-dimensional non-local density functional theory assuming heterogeneous surfaces (2D-NLDFT). Inner sphere diameters and wall thicknesses were determined from TEM micrographs by the average of 10 spheres manually measured with ImageJ.^21^

For the collection of Raman spectra in the spectral range between 100 cm^−1^ and 3500 cm^−1^, a 532 nm laser excitation wavelength and a laser power of 4 mW on the sample were adjusted. A Thermo Scientific DXR2 Raman microscope was equipped with a confocal microscope BX41 (Olympus Corp.) and a 10× objective (numerical aperture of 0.25), delivering a laser spot diameter of approximately 2.1 μm. Therefore, a laser intensity on the sample of about 1.2 mW μm^−2^ is obtained. The full range grating with 900 lines mm^−1^ together with a 50 μm pinhole-like entrance slit to the spectrometer yielded a spectral resolution (equivalent to the full-width half maximum of the instrumental line width) of about 1 cm^−1^.

The X-ray photoelectron spectroscopy (XPS) measurements were performed using the K-Alpha XPS System from Thermo Scientific. The photon source was a monochromatized Al-Kα line (1486.6 eV). The spectra were acquired using a spot size of 400 μm and constant pass energy. A combined low-energy electron/ion flood source was used for charge neutralization.

The self-standing electrodes employed for electrochemical measurement contained 90 mass% of carbon spherogel powder, 5 mass% of conductivity additive (Super C65, Imerys), and 5 mass% of binder (polytetrafluoroethylene, 60 mass% dispersion in water, Sigma Aldrich). The three components were rigorously mixed with a small amount of isopropanol (Avantor, 99.5%) to prepare a homogenous slurry. The obtained dough was calendared within a rolling machine (HR01 hot rolling machine, MTI) to form a sheet of carbon electrode with a thickness of ∼200 μm. Next, the carbon sheet was vacuum-dried for 12 h at 120 °C to remove the remaining solvent. When assembling the cell for electrochemical characterizations, 12 mm diameter round disks were cut and arranged as a symmetric two-electrode set up in custom-built polyether ether ketone (PEEK) cells with spring-loaded titanium pistons.^22^ The glass fiber mat discs (GF/A, Whatman) with a diameter of 13 mm were used as the separator, and 1 M KOH solution in Milli-Q water was used as the electrolyte.

For the electrochemical characterizations, the PEEK cells were tested inside a climate chamber set at 25 ± 1 °C. The tests were performed using a VSP3 potentiostat/galvanostat (Biologic). The investigations were conducted by cyclic voltammetry (CV) at different scan rates ranging from 5–250 mV s^−1^, galvanostatic charge/discharge (GC/GD) at specific currents from 200 mA g^−1^ to 5 A g^−1^, and electrochemical impedance spectroscopy (EIS) at 0.0 V with a sinusoidal signal of 5 mV s^−1^ in the frequency range from 100 kHz to 1 mHz.

Small-angle scattering experiments used a Bruker N8-Horizon (Bruker AXS). The scattering data were integrated to result in functions of scattering intensity *I*(*q*) as a function of the absolute value of the scattering vector *q* = 4π/*λ*·sin(*θ*/2), where *λ* is the wavelength of the radiation used and *θ* the scattering angle. These scattering curves were corrected for transmission effects, and instrumental and constant backgrounds were subtracted. The data were analyzed in two ways: an indirect Fourier transformation was calculated to result in a thickness pair distance distribution function *p*_t_(*r*) of the shell,^23,24^ where *r* is a distance within the shell. In addition, the high *q* part of the scattering curves was approximated by a model that combines a Guinier approximation^25^ for microscopic pores with a polydisperse spherical shell.

The vario micro cube system from elementar was used to perform elemental analysis (CHNS-O). Each sample was weighed in tin boats with a consistent amount of WO_3_ and pressed while excluding air. The combustion tube temperature was set at 1150 °C, and the reduction temperature in the pipe was at 850 °C. The device was calibrated by taking repeated measurements of sulfanilamide. Using a rapid OXY cube oxygen analyzer from elementar, elemental oxygen was quantitatively analyzed at a pyrolysis temperature of 1450 °C. The samples were weighed in silver boats and pressed while excluding air. The system was calibrated by conducting measurements of benzoic acid.

## Results and discussion

### Parameter sets for the IPA-based sol–gel process of carbon spherogels

**Fig. 1:**
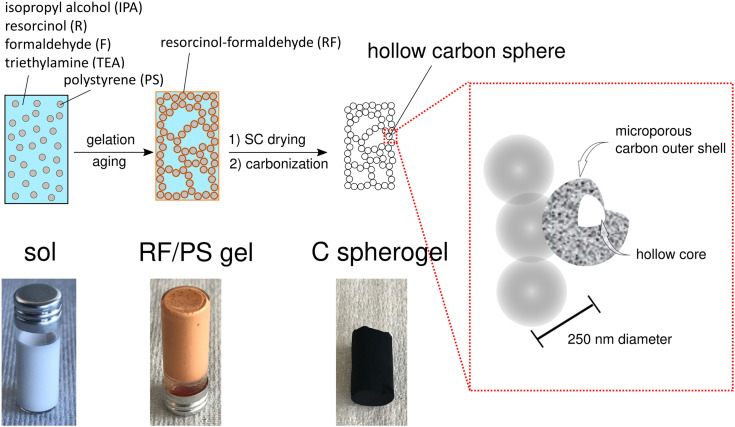
Schematic overview of the sol–gel process from IPA-based sol to a resorcinol–formaldehyde/polystyrene (RF/PS) gel to a carbon spherogel.

First, suitable parameter sets were explored for preparing carbon spherogel structures by IPA-based sol–gel processing. The general synthesis steps are schematically summarized in Fig. 1. The first step in IPA-based sol–gel processing using PS sphere templates consists of gelation and aging of the sol due to a substitution and polycondensation reaction analogously to the water-based sol–gel process.^7^ Resorcinol and PS, dissolved in IPA, can interact two-fold *via* an electron orbital overlap: first by π-stacking of their phenol rings and second by the interaction of the partially charged resorcinol molecule (due to the presence of two OH^−^ groups) with the negatively charged PS surface (due to the presence of sulfate groups). These interactions lead to an efficient molecular coating of all PS spheres with an RF network. The second step of the conventional sol–gel process to carbon spherogels exploits supercritical drying of the RF/PS organogels with carbon dioxide (at 110 bar and 60 °C), followed by the carbonization under an inert atmosphere at 800 °C (which typically is associated with a mass loss of 30 mass%). Corresponding photographs are shown in Fig. 1.

Unlike water-based systems, the typically used polymerization catalyst, namely sodium carbonate, was unsuitable due to its insolubility in IPA. Instead, four candidates for replacement were identified, which are soluble in organic solvents and were described already as potential catalysts for the gelation of resorcinol–furfural in IPA:^15^ sodium hydroxide (NaOH), hydrochloric acid (HCl), hexamethylenetriamine (HMTA) and triethylamine (TEA). TEA yielded complete solubility, the visually most homogeneous gel, and was used as the catalyst in the following experiments (Fig. S1, ESI†).

**Fig. 2:**
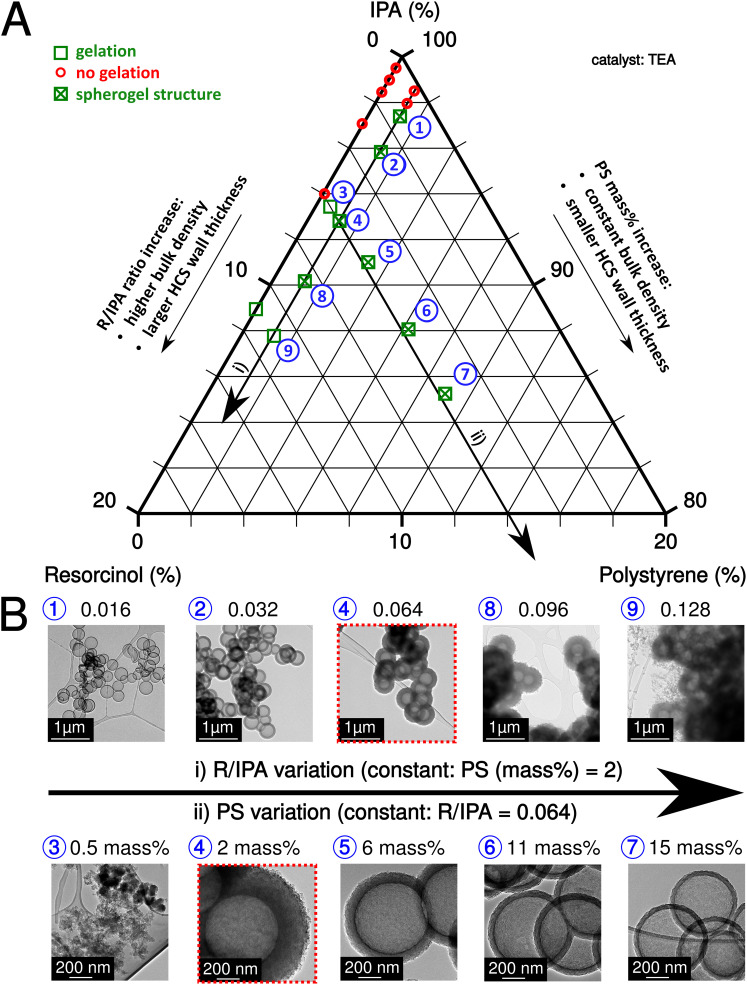
Ternary diagram of the system IPA, PS, and R (A). The arrows indicate the two possibilities to tailor the HCS wall thickness. The green cross marks the parameter combination which yields spherogel structures. Transmission electron micrographs depicting carbon spherogels derived from the two possible methods to tailor the HCS wall thickness (B): (i) R/IPA variation and (ii) PS variation. Analogous samples are marked in red-dashed boxes.

The sol–gel process of IPA-based carbon spherogels mainly depends on the interplay of three component ratios: IPA, resorcinol, and PS (amounts of formaldehyde were kept constant with a ratio (R/F of 0.5)). The resorcinol to initiator (R/TEA) molar ratio was also fixed at 10 (relatively low according to the literature^26^) to increase the number of seed particles for cluster formation and to exclude it as a limiting factor for gelation. Fig. 2A summarizes this system visualized in a ternary diagram: As indicated by the two black arrows, fortunately, various parameter conditions are found appropriate for spherogel preparation the R/IPA molar ratios were varied in the range from 0.004 to 0.128, while the PS concentration in IPA (PS amount) from 0 to 15 mass%. Our synthesis allows tailoring the hollow carbon sphere's wall thickness deliberately.

The variation of the molar ratio of resorcinol to IPA or the variation of the PS concentration in IPA can achieve the abovementioned tailoring. In the first case, the wall thickness increases by adding more resorcinol and *vice versa*. However, the bulk density increases simultaneously as well. In the second case, more nanospheres are coated by increasing the PS concentration (and keeping the R/IPA ratio constant), decreasing the wall thickness. Fig. 2B displays the corresponding transmission electron micrographs of carbon spherogels and the effect of the R/IPA ratio and PS concentration in IPA as described. The minimum PS concentration in IPA was identified in the 1–2 mass% range.

As expected, carbon spherogels prepared from the lowest R/IPA ratios yielded the lowest wall thicknesses. The first gelation occurred at R/IPA = 0.016 at 2 mass% PS. Adding resorcinol–formaldehyde precursors provided more material and increased the wall thickness (18 ± 2 nm to 226 ± 11 nm). Analogically, providing more PS templates decreased the wall thickness. At a R/IPA ratio of 0.064 with 2 mass%, the wall thickness decreased from 132 ± 6 nm to 21 ± 1 nm at 15 mass% PS.

For comparison, a sol containing no PS spheres was prepared. Gelation occurred for the first time at R/IPA = 0.128. In contrast, a sol containing 2 mass% PS spheres gelled starting from R/IPA = 0.016. A sol containing 0.5 mass% PS was sufficient to promote gelation at 0.064 R/IPA but yielded no spherogel structures. **Table S1** (ESI†), summarizes the investigated combinations, the resulting wall thicknesses, specific surface areas, and pore volumes.

For a detailed investigation of the pore structure of different wall thicknesses, depending on the applied sphere templating amount, we selected the sample series with a fixed R/IPA ratio of 0.064. Nitrogen adsorption experiments were conducted, and the respective isotherms and quantitative results are shown in **Fig. S2** and **Table S1** (ESI†). Thin-walled samples (15 mass% PS) show a typical hysteresis loop for hollow spherical samples (H2-type hysteresis due to cavitation; ref. 27). Samples prepared with a lower PS concentration do not show a hysteresis loop, which can be explained by a dense wall texture that prevents the diffusion of nitrogen gas and the filling of spheres with nitrogen. A rougher hollow carbon sphere wall texture is visible compared to water-based systems. The pore size distribution indicates that most of the pores are smaller than 1 nm, compared to the presence of 1–2 nm pores in water-based carbon spherogels (**Fig. S2B**, ESI†).^9^

### Simplification of the drying process of RF/PS spherogels: direct carbonization *versus* SC-drying and carbonization

**Fig. 3:**
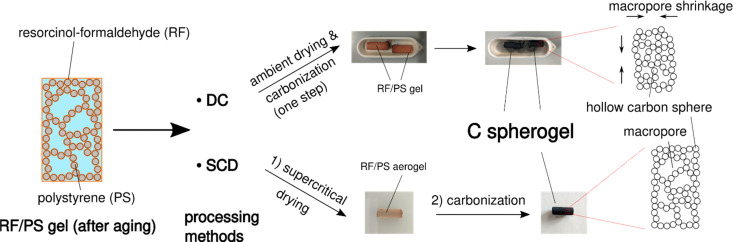
Schematic overview of the two main options to convert an RF/PS gel into a carbon spherogel. The processing of RF/PS gels can either follow a DC or SCD route. The photographs show gels before and after carbonization.

Supercritical drying is probably the critical processing step in carbon aerogel/spherogel preparation, which appears at the same time as time- and energy-consuming and dependent on expensive equipment. Realizing an additional benefit of using IPA as the pore solvent with the result of a lower surface tension compared to deionized water, ambient drying could be a promising option. This feature allows the investigation of the potential for direct carbonization of a wet gel. Fig. 3 depicts the synthesis scheme of carbon spherogels by direct carbonization (DC) compared to a classical way based on supercritical drying and carbonization carbonized (SCD).

To also evaluate the influence of different wall thicknesses on both pathways (DC and SCD), two carbon spherogel variants were prepared, one with thin sphere walls (approx. 35 nm; labeled DC_35 and SCD_35) and one with thick walls (approx. 125 nm; labeled DC_125 and SCD_125). As described in the previous section, a careful adjustment of the sphere walls could be achieved by the following parameters: an R/IPA ratio of 0.064 (R/F was set to 0.5), an IPA/PS solution containing 6 mass% PS (for the 35-nm-walled) and 1.5 mass% PS for the 125-nm-walled sample. As depicted by the corresponding nitrogen sorption isotherms in **Fig. S4** (ESI†), direct carbonization (DC) slightly lowers the micropore content compared to SCD processing.

### Post-synthetic physical activation with carbon dioxide

**Fig. 4:**
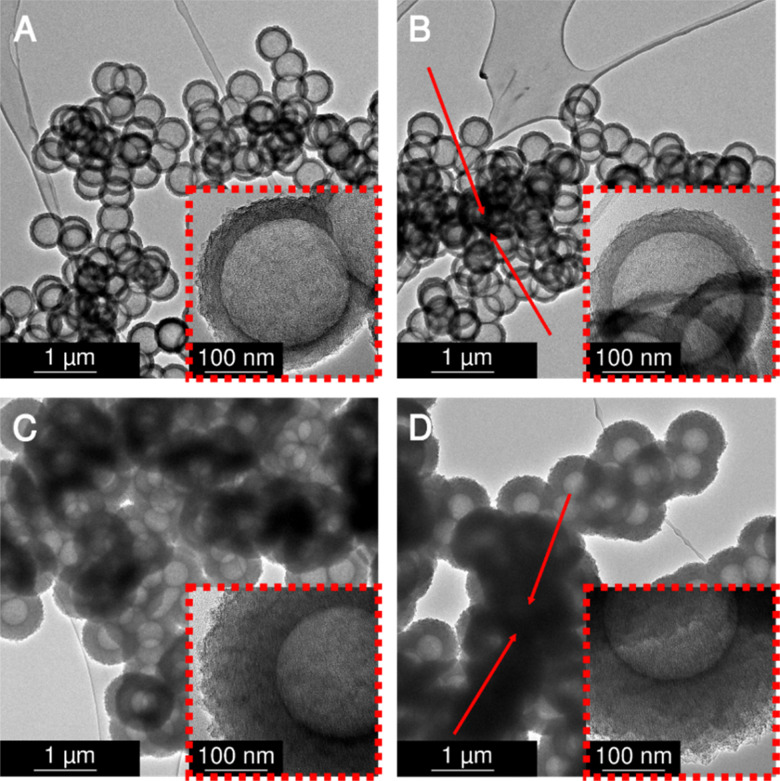
Transmission electron micrographs of DC and SCD carbon spherogel samples after physical activation with CO_2_. (A) 35-nm-walled sample, SCD; (B) 35-nm-walled sample, DC; (C) 125-nm-walled sample, SCD; (D) 125-nm-walled sample, DC.

Subsequently, the four variants were physically activated with CO_2_ at 900 °C for 2 h to enlarge the micropore content and increase the specific surface areas. Transmission electron micrographs (Fig. 4) analysis shows no damage to the hollow carbon sphere nanostructure. However, a distinct macropore shrinkage (pertaining to the decrease of the diameter of the hollow core inside the spherogel spheres) in directly carbonized samples is visible. Fig. 4 compares the carbon spherogel morphology of DC-treated and SCD-treated organogels. **Fig. S3** (ESI†), shows transmission electron micrographs before activation for comparison. Fortunately, both synthetic variants show a perfect hollow sphere morphology before and after physical activation. However, the bulk diameter of the DC carbon spherogel monolith was 8% smaller than the SCD carbon spherogel (9.2 mm *vs.* 10 mm). This higher linear shrinkage indicates a distinct macropore loss during direct carbonization. The freestanding bulk of the DC carbon spherogel noticeably displays increased brittleness due to the lower amount of macropores compared to an SCD carbon spherogel.

Nitrogen sorption isotherms were recorded before and after CO_2_ activation. The results after activation are summarized in Table 1 and before activation in **Table S1** (ESI†). In their pristine state, non-activated, DC carbon spherogels show a slightly lower specific surface area (DC_125 sample: 749 m^2^ g^−1^, DC_35 sample: 697 m^2^ g^−1^) compared to SCD carbon spherogels (SCD_125 sample: 1072 m^2^ g^−1^, SCD_35 sample: 820 m^2^ g^−1^). This deviation can be pore densification and, therefore, loss of surface area caused by solvent evaporation (in contrast to surface tension-free supercritical extraction with CO_2_ of the SCD samples). The respective isotherms are depicted in **Fig. S4** (ESI†). No sample showed a pronounced hysteresis loop, indicating that the carbon wall did not allow diffusion of nitrogen gas into the sphere interior.

**Table 1:** Specific surface areas (SSA) and pore volume measurements relating to the wall thickness of activated carbon spherogels, 125-nm-walled and 35-nm-walled variants

Sample	SSA (2D-NLDFT)	Inner sphere diameter^a^ (TEM)	Wall thickness^a^ (TEM)	N-content	Total pore volume (2D-NLDFT)	Micropore volume (2D-NLDFT)	Average pore size d_50_ (2D-NLDFT)	Micropore diameter (SAXS)
	(m^2^ g^−1^)	(nm)	(nm)	(mass%)	(cm^3^ g^−1^)	(cm^3^ g^−1^)	(nm)	(nm)
SCD_35	3323	213 ± 6	35 ± 2	2.4 ± 0.5	1.37	1.04	0.70	0.90 ± 3.8
DC_35	3584	209 ± 5	34 ± 3	2.4 ± 0.8	1.44	1.14	0.70	0.87 ± 4.7
SCD_125	3672	210 ± 5	124 ± 5	2.2 ± 0.6	1.36	1.17	0.68	0.85 ± 5.4
DC_125	2308	211 ± 4	126 ± 4	3.4 ± 0.8	0.70	0.75	0.61	0.85 ± 6.0

a Average of 10 measurements.

**Fig. 5:**
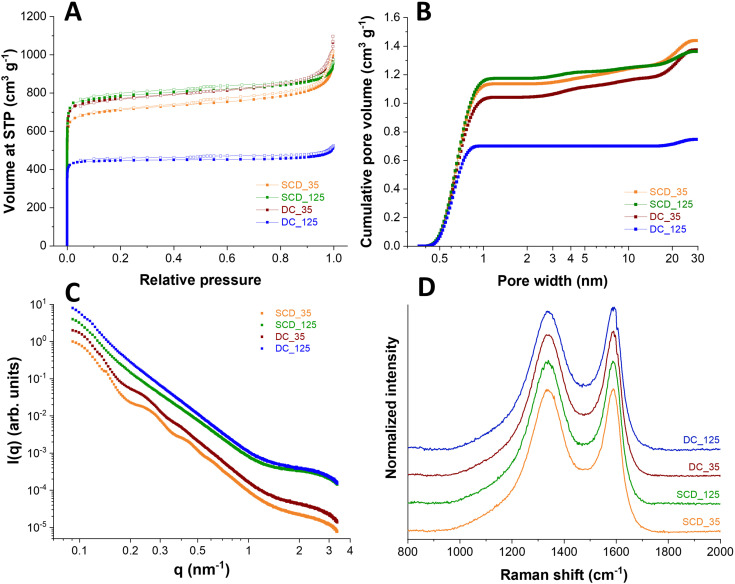
Structural analysis of DC_125; DC_35, SCD_125, SCD_35. Nitrogen sorption isotherms (A) and pore size distribution curves (B) of directly carbonized (DC) and first supercritically dried and then carbonized (SCD) samples after physical activation with CO_2_ at 900 °C for 90 min. (C) SAXS scattering curves; (D) Raman spectra of SCD and DC samples after CO_2_ activation (samples are shifted vertically for better visibility).

Physical activation with carbon dioxide at 900 °C was applied to tune the sphere wall porosity further. Thereby, the specific surface areas were increased to 3300–3670 m^2^ g^−1^ for all samples except DC_125 carbon spherogel. During activation, new micropores are generated, and the samples are homogenized. The DC_125 samples show a minimized diffusion of CO_2_ and lowered accessibility of the sphere walls during the activation process, as we used monolithic samples in this step; thus, the resulting SSA shows only 2308 m^2^ g^−1^ (Fig. 5A). The pore size distribution shows an average pore size of approximately 0.6 nm micropores for all samples (Fig. 5B).

Small angle scattering (SAXS) was applied to the samples to confirm the structural properties. The SAXS curves of the samples (Fig. 5C) show a general decay proportional to 1 *q*^−2^ due to the shell thickness being considerably smaller than the diameter of the spheres. This results in the observed low curvature. This feature is modified for samples SCD_35 and DC_35 by oscillations, which are related to the thickness of the shell, which was found to be 39 nm for sample SCD_35 and 42 nm for sample DC_35. The oscillations are weaker for sample DC_35, indicating larger polydispersity, and absent for samples SCD_125 and DC_125 due to even greater polydispersity. Quantitative analysis leads to coefficients of variation of 10% (SCD_35), 11% (DC_35), 13% (SCD_125), and 14% (DC_125). This polydispersity in shell thickness can also be due to shells of varying thickness for each sphere. All samples show a shoulder at large *q*-values due to small structures, most likely micropores. The more pronounced shoulders in the scattering curves of samples SCD_125 and DC_125 show that the amount of these micropores is higher by about a factor of two relative to sample SCD_35. At the same time, the average pore radius is slightly reduced, as can be seen from the results of the model approximation. These findings are supported by the independent indirect Fourier transformation (**Fig. S5**, ESI†). The mostly linear decay shows the low curvature and polydispersity of sample SCD_35. The thickness can be estimated from the point where the curve reaches zero. A slight oscillation at small distances can be attributed to micropores. Sample DC_35 is similar, but the function is not as straightforward due to higher polydispersity. This is even more the case for samples SCD_125 and DC_125, where the oscillations at small distances are more pronounced due to a larger number of structures of a size of about 1 nm. As listed in Table 1, the SAXS measurements confirm similar micropore diameters for all samples at 0.85–0.90 nm. After activation, only a minimal amount of pores larger than 1 nm is generated (<2 cm^3^ g^−1^).

For electrochemical evaluation, a suitable electrolyte system that can diffuse into the micropores and the interior sphere void has to be identified to take advantage of the benefits of hollow carbon sphere structures. The average micropore size and total micropore volume can be increased through longer physical activation in CO_2_, more prolonged carbonization times, or a higher R/TEA ratio. Table 1 summarizes the TEM evaluation of the wall thickness, specific surface area, and pore size distribution data obtained from gas physisorption.

The samples were further characterized by Raman spectroscopy. All Raman spectra (Fig. 5D) displayed for the carbon materials, both a characteristic D-band and G-band contribution (at 1342 cm^−1^ and 1591 cm^−1^, respectively), indicating the presence of disordered, nanocrystalline, sp^2^-hybridized carbon (D-band: A_1g_ in-plane breathing mode; E_2g_ in-plane vibration mode).^28,29^ By calculating the A_D_/A_G_ ratio of D-band and G-band areas, a measure for the degree of graphitization is obtained. Similar values between 2.3 and 2.5 for all samples were calculated, which suggests an incomplete crystalline character with distinct contributions of amorphous carbon. Peak deconvolution was applied to the recorded spectra assuming five band components, namely D*, D, D**, G, and D′ (**Fig**. **S6**, ESI†). Assuming this number of bands, which align with literature works on incompletely graphitic materials,^30–32^ we have obtained an excellent fit of the experimentally measured spectra. As seen from **Fig. S6** (ESI†), all carbons show very similar spectra, and this similarity extends when comparing the fitted contributions of the assumed five band components. These similarities extend also to the set of defect-activated bands.

**Fig. 6:**
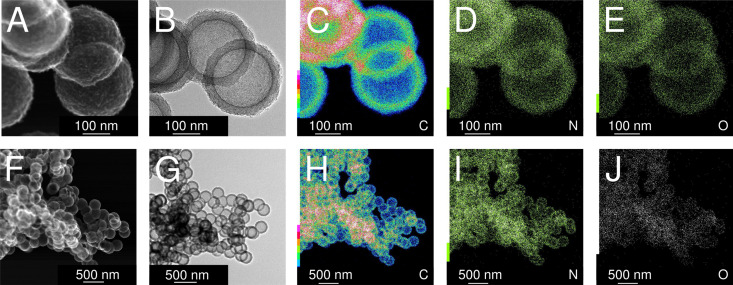
Scanning transmission electron micrographs (A) and (F), transmission electron micrographs (B) and (G) and elemental maps using STEM-EDX (for the elements carbon (C) and (H), nitrogen (D) and (I), and oxygen (E) and (J)) of activated carbon spherogels, analogous to sample (4) in Fig. 2.

As a consequence of using TEA as the gelation catalyst, IPA-based carbon spherogels are doped with nitrogen. Nitrogen is detected throughout the sample and is homogeneously distributed. Fig. 6 displays transmission electron micrographs of a typical carbon spherogel sample (sample (4) in Fig. 2) following the IPA-based process. Quantitative insights of the nitrogen contents were elucidated by CHNS-O analysis, indicating a rather similar amount of 2.2–3.4 mass% (Table 1). These observations were confirmed by X-ray photoelectron spectroscopy: all carbon-dioxide-activated carbon spherogel samples, either supercritically dried (SCD_35 or SCD_125) or directly carbonized (DC_35 or DC_125) show very similar survey scans for C (285 eV), N (395–405 eV) and O (533 eV) (**Fig. S7**, ESI†). Besides the general low nitrogen content and the very low signal intensity, XPS data seem to indicate the presence of pyridinic and graphitic nitrogen (**Fig. S7C**, ESI†).^28,33,34^

### Electrochemical testing

**Fig. 7:**
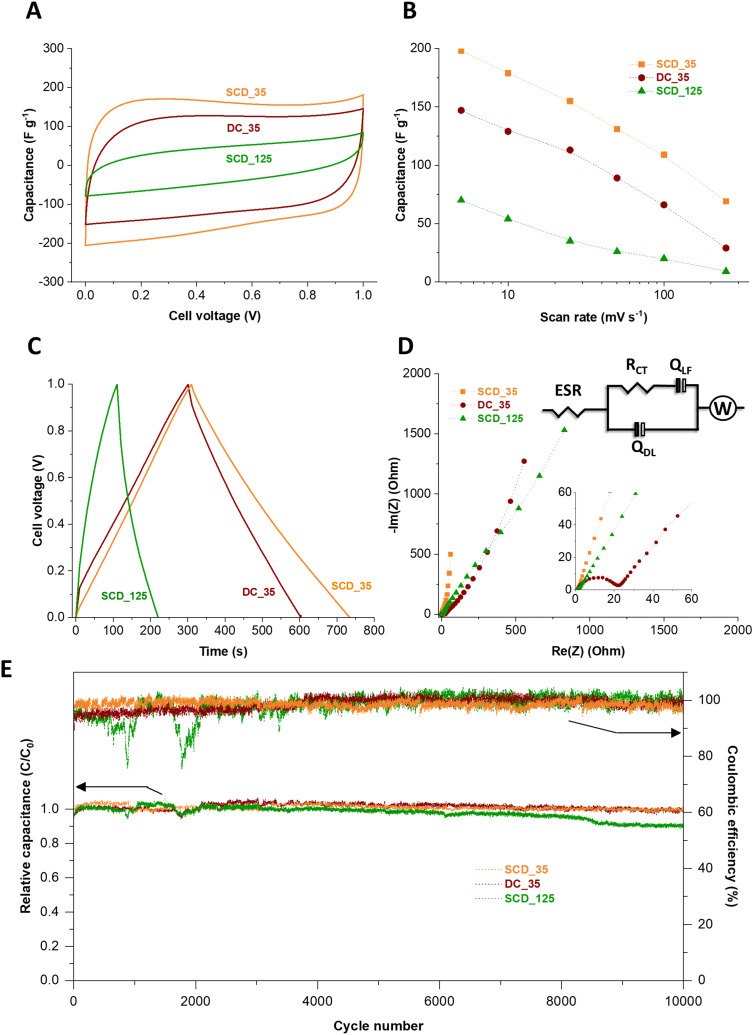
Electrochemical performance of different carbon spherogels (SCD_35, DC_35, and SCD_125) operated in a symmetric configuration as a supercapacitor in 1 M KOH aqueous electrolyte. (A) 5th cycle of different synthesized carbon spherogels in cyclic voltammetry recorded at a scanning rate of 10 mV s^−1^, (B) specific capacitance *vs.* scan rate, (C) charge and discharge showing voltage profiles of DC_35, SCD_35, and SCD_125 at 200 mA g^−1^, and (D) Nyquist plots of different carbon spherogels (SCD_35, DC_35, and SCD_125) operated in a symmetric configuration as a supercapacitor in 1 M KOH aqueous electrolyte. The inset represents the magnification of the high-frequency region and the corresponding equivalent circuit of the plots. (E) Electrochemical stability performance showing the relative capacitance retention and the Coulombic efficiency of different carbon spherogel applying a specific current of 200 mA g^−1^ for 10 000 cycles.

The electrochemical properties of the 125-nm-walled and 35-nm-walled carbon materials in 1 M KOH aqueous electrolyte have been studied by CV, GC/GD, and EIS. All samples showed an overall cyclic voltammetric pattern typical for electrical double-layer capacitors (**Fig. S8**, ESI†).^35^ SCD_125, the material with the overall lowest specific surface area (2308 m^2^ g^−1^) and lowest pore volume (0.70 cm^3^ g^−1^), has the poorest electrochemical performance with less than 20 F g^−1^ even at a very low scan rate of 5 mV s^−1^.^36^ A high surface area and accessible pore volume is instrumental to obtaining high specific capacitance *via* ion electrosorption. We therefore focus the following investigation more on the three materials with higher specific surface area, pore volume, as seen from Fig. 7A, B and **Fig. S8** (ESI†), a much higher specific capacitance, namely DC_35, SCD_35, and SCD_125. While all samples show a rather similar specific surface area (3323–3672 m^2^ g^−1^), average pore size (0.68–0.70 nm), and total pore volume (1.36–1.44 cm^3^ g^−1^), there are significant differences between the materials when comparing the specific capacitance values. For example, at a slow rate of 5 mV s^−1^, we see the highest capacitance for SCD_35 (198 F g^−1^), followed by DC_35 (147 F g^−1^), and concluded by SCD_125 (70 F g^−1^). As will be seen later, the latter sample also shows the most resistive behavior.^37,38^

This sequence is also confirmed by galvanostatic charge/discharge cycling (Fig. 7C). The specific capacitance obtained from GC/GD cycle at 500 mA g^−1^ are 204 F g^−1^, 156 F g^−1^, and 58 F g^−1^ for the cells based on SCD_35, DC_35, and SCD_125 electrodes, respectively. Accordingly, the volumetric capacitances of the cells were calculated by measuring the electrode densities. As a result, the corresponding volumetric capacitance values are 39 F cm^−3^, 37 F cm^−3^, and 12 F cm^−3^ for SCD_35, DC_35, and SCD_125 cells, respectively. The Coulombic efficiency of the devices made with SCD_35 and DC_35 electrodes neared 100%, compared to only 83% for the SCD_125-based cell.

When comparing the carbon spherogels with the thin shell, we see a more resistive behavior for DC_35 compared to SCD_35. This can be inferred, for example, from the larger iR drop (Fig. 7C and **Fig. S9**, ESI†) and the electrochemical impedance spectra.

The higher capacitive properties of the SCD_35 electrode are attributed to its distinctive pore structure, as elucidated by SAXS analysis (Fig. 5C). Although exhibiting a slightly lower specific surface area than DC_35 and SCD_125 carbon spherogels (Table 1), SCD_35 displays a low curvature and polydispersity, indicating a well-defined micropore structure – beneficial for superior EDL charging. The Nyquist plots and the corresponding equivalent are shown in Fig. 7D. This circuit take considers physical phenomena occurring at the interface between electrode and electrolyte. In the model, the high-frequency range is analyzed through the ESR (equivalent series resistance), *R*_ct_ (charge transfer resistance), and *Q*_dl_ (constant phase element of the capacitance of the double layer). The lower frequency range was investigated with *Q*_lf_ which is a constant phase element related to the capacitance of charge intercalation into the spherogel structure and a Warburg element to account for the diffusion resistance of the system.

The impedance spectra suggest a capacitive behavior of the cells, where the device made of SCD_35 electrode is closer to the ideal behavior, displaying a straight line almost parallel to the imaginary axis in the low frequency region. At the higher frequencies, the Nyquist plots indicate an increase in the equivalent series resistance from 0.63 Ω to 0.70 Ω and 0.97 Ω for the SCD_35, DC_35, and SCD_125 cells, respectively, suggesting a distinction on the interfacial properties of the electrodes. Furthermore, the kinetic behavior of the materials has a significant distortion on diffusional characteristics of the samples, which may be an effect caused by interfacial interactions promoted by different surface termination groups of each sample. The data for SCD_35 suggest a higher adsorption/desorption of the electrolyte onto the surface of the electrode, while the other two samples indicate an entrapment of charges allowing intercalation onto the pores, hindering the diffusional interactions. This is supported by the change on the capacitive behavior of the different samples, further indicating the effect of surface groups over the electrochemical kinetic performance of the materials. The performance stability was tested for 10 000 galvanostatic charge/discharge cycles.^39^ In Fig. 7E, it can be seen that the relative capacitance of the cells slightly fluctuates during the first hours of cycling due to the improved penetration of ions in the tortuous porosity of the materials.^40–42^ From 3000 to 10 000 GC/GD cycles, the capacitance is relatively stable for the device made with SCD_35 and DC_35 electrodes. For the cell based on SCD_125, the capacitance continuously decreases to 89% of its initial value after 10 000 GC/GD cycles. After 10 000 GC/GD cycles, the relative capacitance of the cells made with SCD_35 and DC_35 electrodes is 100% and 97%, respectively, and the Coulombic efficiency of the three cells is maintained at 100%. The measured stability is very high considering the device failure margin of 20% capacitance loss commonly used in applications and industry.^22,39,43^

Comparing the electrochemical performance obtained by applying optimized carbon spherogels as supercapacitor electrode material with state-of-the-art literature values, these materials synthesized and tested in this work can keep up with the average of different reported literature values. In Table 2, a summary of different electrochemical performances and parameters of several asymmetric and symmetric supercapacitors collected from the literature are reported and compared. Compared to other symmetric carbon-based systems like the porous graphitic biomass carbon explored by Gong *et al.*^44^ we obtained more than double the capacitance with the double specific current and reported cycles. This system reports a specific capacitance of 100 F g^−1^ recorded at 100 mA g^−1^ for 5000 cycles, while SCD_35 achieves a capacitance of 204 F g^−1^ at 200 mA g^−1^ for 10 000 cycles. Li *et al*. established in 2017 a self-supporting activated carbon/carbon nanotube/reduced graphene oxide flexible electrode for asymmetric supercapacitor application while achieving a high capacitance of 195 F g^−1^ with a higher specific current of 5000 mA g^−1^ for 5000 cycles with a capacitance retention of 93.7%.^45^

**Table 2:** Overview of electrochemical performance and parameters of different asymmetric and symmetric supercapacitors in aqueous (aq), organic (org), or ionic liquids (IL) electrolytes. Not available data from literature references are donated as “n.a.”

Ref.	Negative electrode	Positive electrode	Surface area (m^2^ g^−1^)	Cell voltage (V)	Electrolyte	Capacitance (F g^−1^)	Specific current (mA g^−1^)	Cycle number	ESR
46	MnO_2_	PEDOT	n.a.	1.8	2 M KNO_3_ (aq)	120	250	500	0.48 Ω cm^2^
55	Cucurbituril subnanoporous carbon	Cucurbituril subnanoporous carbon	745	3.8	MMIMBF_4_ (IL)	234	200	20 000	15.8 Ω
44	Porous graphitic biomass carbon	Porous graphitic biomass carbon	1732	3.5	EMIMBF_4_ (IL)	100	100	5000	n.a.
56	VS_4_ nanostructures on carbon fiber cloth	Pt	n.a.	2.0	EMImOTf (IL)	206	1000	1000	3.12 Ω cm^2^
47	Rod-like porous carbon	Nickel sheet	1610	0.9	6 M KOH (aq)	222	100	10 0000	0.76 Ω
57	Nanoporous carbon	Nanoporous carbon	1260	1.0	6 M KOH (aq)	169	1000	10 000	n.a.
45	Nitrogen-doped porous carbons	Carbon rods	1052	1.0	2 M KOH (aq)	205	5000	5000	n.a.
9	Monolithic carbon spherogels	Monolithic carbon spherogels	1931	2.5	1 M TEABF_4_ in ACN (org)	125	1000	10 000	n.a.
53	Polystyrene/polyacrylonitrile nitrogen doped hollow mesoporous carbon spheres	Polystyrene/polyacrylonitrile nitrogen doped hollow mesoporous carbon spheres	955	1.0	6 M KOH (aq)	406	2000	10 000	n.a
49	Carbon spheres pyrolyzed from polystyrene@polypyrrole-polyaniline	Carbon spheres pyrolyzed from polystyrene@polypyrrole-polyaniline	354.27	0.9	1 M KOH (aq)	186	500	5000	n.a.
50	Monodispersed N-doped carbon nanospheres	Monodispersed N-doped carbon nanospheres	145.5	1.0	1 M H_2_SO_4_ (aq)	191	1000	10 000	n.a.
51	Hollow mesoporous carbon spheres	Hollow mesoporous carbon spheres	1321	1.0	6 M KOH (aq)	157	500	5000	2.8 Ω
48	Porous carbon derived from waste polystyrene foam	Porous carbon derived from waste polystyrene foam	620	1.0	6 M KOH (aq)	208	1000	5000	0.34 Ω
52	Polystyrene-based carbon spheres	Polystyrene-based carbon spheres	996	1.0	6 M KOH (aq)	182	—	—	—
This work	SCD_35	SCD_35	3323	1.0	1 M KOH (aq)	204	200	10 000	0.63 Ω

Compared to other electrode materials reported in the literature, the optimized carbon spherogels synthesized in this work exhibit competitive electrochemical performance. For instance, a supercapacitor based on a positive amorphous manganese dioxide (MnO_2_) electrode and negative poly(3,4-ethylenedioxythiophene) (PEDOT)^46^ one was reported with a capacitance of 120 F g^−1^ at 250 mA g^−1^ for 500 cycles with an ESR of 0.48 Ω cm^2^, while rod-like porous carbon achieved a capacitance of 222 F g^−1^ at 100 mA g^−1^ for 100 000 cycles with an ESR of 0.76 Ω.^47^ In comparison, our SCD_35 sample achieved a capacitance of 204 F g^−1^ at 200 mA g^−1^ for 10 000 cycles with an ESR of 0.63 Ω (0.80 Ω cm^2^), indicating competitive performance.

We evaluated the SCD_35 sample against various carbon spheres and structures and observed that our monolithic carbon spherogels outperform several counterparts. For instance, in comparison to porous carbon derived from waste polystyrene foam with a capacitance of 208 F g^−1^ and cycled for 5000 cycles,^48^ the SCD_35 sample demonstrates a competitive capacitance of 204 F g^−1^ and cycled for 10 000 cycles, emphasizing its potential for long-term electrochemical stability. Additionally, our SCD_35 sample showcases better performance compared to other materials, including carbon spheres pyrolyzed from polystyrene@polypyrrole-polyaniline,^49^ monodispersed N-doped carbon nanospheres,^50^ hollow mesoporous carbon spheres,^51^ and polystyrene-based carbon spheres.^52^

Overall, the optimized samples from this work achieve the literature values reported in state-of-the-art compared to symmetric and asymmetric supercapacitor systems in the aqueous, organic, and ionic-liquid-based electrolytes.^9,47,53–56^ For electrochemical applications in supercapacitors, we see the importance of the high specific surface area and carbon porosity, which can be further tuned in the following studies.

## Conclusions

The sol-gel process of carbon spherogels was successfully replicated in IPA. While a pH adjustment is impossible in organic solvents, the careful design of micro- to macropore structure can be adjusted by R/IPA (bulk density) and R/TEA (degree of seed particle generation) molar ratios. However, the preparation of reversibly compressible samples remains challenging. The critical parameter combinations (amount of IPA, R, and PS) were documented in a ternary diagram. In contrast to water-based variants, carbon spherogels made in IPA show a denser carbon wall texture. The PS amount and the R/IPA ratio can adjust the wall thickness. The ideal R/IPA ratio was 0.064 at 2 mass% due to the relatively high wall thickness, which can be fine-tuned *via* the PS concentration.

The IPA-based sol–gel process of carbon spherogels gives two main advantages compared to the water-based analogs: (1) time and material costs can be reduced *via* direct carbonization of wet gels as the optimized ambient solvent removal and pyrolysis only require one step. Ambient drying is facilitated due to IPA's lower surface tension than water. DC samples measured a lower specific surface area in their pristine state after carbonization (approximately 600 m^2^ g^−1^*vs.* 800 m^2^ g^−1^ of supercritically dried and carbonized variants). However, after physical activation with CO_2_, the surface areas could be increased for all samples above 3300 m^2^ g^−1^, except a thick-walled variant (125 nm; 2300 m^2^ g^−1^). Process optimization (drying time in the carbonization oven, optimal wall thickness, and R/TEA ratio) is essential for further research on cost-efficient carbon spherogel preparation. (2) The organic solvent facilitates the preparation of homogeneous hybrid carbon spherogel variants since many metal oxide salts (for instance, metal acetylacetonates) can only be dissolved in organic solvents. This possibility opens the door to new application fields in energy storage systems that require hybrid carbon materials (*e.g.*, carbon as catalyst support for oxygen reduction reactions in fuel cells).

The electrochemical results of supercapacitors made with the different materials showed that the SCD_35 carbon spherogels show higher capacitive properties than the DC_35, SCD_125, and DC_125 carbon spherogels, owing to its higher micropore volume and specific surface area. Accordingly, the cells made with SCD_35 and DC_35 electrodes showed good stability performance, with capacitance retention maintained at 100% and Coulombic efficiency at 100% after 10 000 cycles at a specific current of 200 mA g^−1^. In comparison, the capacitance of the cell made with SCD_125 decreased to 89% of its initial value after 10 000 cycles.

The unique feature of carbon spherogels, the combination of hollow carbon sphere network units with the high porosity and surface area of nanoporous carbons, will be the main focus of further investigations. In typical porous carbon materials, macropore shrinkage after ambient drying would inhibit the filling/wetting of electrolytes as the material would be too dense.

## Author contributions

MS: conceptualization, synthesis, investigation, data curation, visualization, writing – original draft. EP: electrochemical investigation, data curation, visualization, writing – original draft. SA: electrochemical investigation, data curation, visualization, review and editing. IS: synthesis, investigation, data curation, visualization. TB: Raman investigation, data curation, validation – review and editing. NH: methodology, investigation, review and editing. GFP: SAXS methodology, investigation, data curation, validation, writing. VP: methodology, investigation, data curation, validation, writing – review and editing. ME: conceptualization, validation, supervision, funding acquisition, writing – review and editing.

## Data availability

Data can be made available upon request by the corresponding author(s).

## Conflicts of interest

No conflicts to declare.

## Supplementary Material

YA-003-D3YA00480E-s001

## References

[cit1] Biener J., Stadermann M., Suss M., Worsley M. A., Biener M. M., Rose K. A., Baumann T. F. (2011). Energy Environ. Sci..

[cit2] Auer E., Freund A., Pietsch J., Tacke T. (1998). Appl. Catal., A.

[cit3] Zhang L., Yao M., Yan W., Liu X., Jiang B., Qian Z., Gao Y., Lu X.-J., Chen X., Wang Q.-L. (2017). Int. J. Nanomed..

[cit4] Liang C., Li Z., Dai S. (2008). Angew. Chem., Int. Ed..

[cit5] Madhu R., Periasamy A. P., Schlee P., Hérou S., Titirici M.-M. (2023). Carbon.

[cit6] Titirici M.-M., White R. J., Brun N., Budarin V. L., Su D. S., del Monte F., Clark J. H., MacLachlan M. J. (2015). Chem. Soc. Rev..

[cit7] Salihovic M., Zickler G. A., Fritz-Popovski G., Ulbricht M., Paris O., Hüsing N., Presser V., Elsaesser M. S. (2019). Carbon.

[cit8] Li S., Pasc A., Fierro V., Celzard A. (2016). J. Mater. Chem. A.

[cit9] Salihovic M., Schlee P., Herou S., Titirici M.-M., Hüsing N., Elsaesser M. S. (2021). ACS Appl. Energy Mater..

[cit10] Liu T., Zhang L., Cheng B., Yu J. (2019). Adv. Energy Mater..

[cit11] Salihovic M., Schoiber J., Cherevan A., Rameshan C., Fritz-Popovski G., Ulbricht M., Arnold S., Presser V., Paris O., Musso M., Hüsing N., Elsaesser M. S. (2021). Chem. Commun..

[cit12] Pekala R. W., Alviso C. (1992). MRS Online Proc. Libr..

[cit13] Elkhatat A. M., Al-Muhtaseb S. A. (2011). Adv. Mater..

[cit14] Fu R., Zheng B., Liu J., Dresselhaus M. S., Dresselhaus G., Satcher Jr J. H., Baumann T. F. (2003). Adv. Funct. Mater..

[cit15] Wu D., Fu R., Zhang S., Dresselhaus M. S., Dresselhaus G. (2004). J. Non-Cryst. Solids.

[cit16] Hüsing N., Schubert U. (1998). Angew. Chem., Int. Ed..

[cit17] Pekala R. (1989). J. Mater. Sci..

[cit18] Guo K., Song H., Chen X., Du X., Zhong L. (2014). Phys. Chem. Chem. Phys..

[cit19] Mulik S., Sotiriou-Leventis C., Leventis N. (2008). Chem. Mater..

[cit20] Du X., He J. (2008). J. Appl. Polym. Sci..

[cit21] Schneider C. A., Rasband W. S., Eliceiri K. W. (2012). Nat. Methods.

[cit22] Weingarth D., Zeiger M., Jäckel N., Aslan M., Feng G., Presser V. (2014). Adv. Energy Mater..

[cit23] Glatter O. (1980). J. Appl. Crystallogr..

[cit24] Glatter O. (1981). J. Appl. Crystallogr..

[cit25] Guinier A. , Fournet G. and Yudowitch K. L., Small-angle scattering of X-rays, John Wiley, 1955

[cit26] Al-Muhtaseb S. A., Ritter J. A. (2003). Adv. Mater..

[cit27] Thommes M. (2010). Chem. Ing. Tech..

[cit28] Wassner M., Eckardt M., Reyer A., Diemant T., Elsaesser M. S., Behm R. J., Hüsing N. (2020). Beilstein J. Nanotechnol..

[cit29] Koopmann A. K., Bartschmid T., Huesing N., Elsaesser M. S. (2023). J. Sol-Gel Sci. Technol..

[cit30] Kaniyoor A., Ramaprabhu S. (2012). AIP Adv..

[cit31] Pimenta M. A., Dresselhaus G., Dresselhaus M. S., Cancado L. G., Jorioa A., Saitoe R. (2007). Phys. Chem. Chem. Phys..

[cit32] Zickler G. A., Smarsly B., Gierlinger N., Peterlik H., Paris O. (2006). Carbon.

[cit33] Kondo T., Guo D., Shikano T., Suzuki T., Sakurai M., Okada S., Nakamura J. (2015). Sci. Rep..

[cit34] Krüner B., Schreiber A., Tolosa A., Quade A., Badaczewski F., Pfaff T., Smarsly B. M., Presser V. (2018). Carbon.

[cit35] Eftekhari A. (2018). ACS Sustainable Chem. Eng..

[cit36] Lee J.-S., Kim S.-I., Yoon J.-C., Jang J.-H. (2013). ACS Nano.

[cit37] Largeot C., Portet C., Chmiola J., Taberna P.-L., Gogotsi Y., Simon P. (2008). J. Am. Chem. Soc..

[cit38] Yambou E. P., Gorska B., Pavlenko V., Beguin F. (2020). Electrochim. Acta.

[cit39] Pameté E., Köps L., Kreth F. A., Pohlmann S., Varzi A., Brousse T., Balducci A., Presser V. (2023). Adv. Energy Mater..

[cit40] Ratajczak P., Jurewicz K., Skowron P., Abbas Q., Béguin F. (2014). Electrochim. Acta.

[cit41] Jäckel N., Weingarth D., Schreiber A., Krüner B., Zeiger M., Tolosa A., Aslan M., Presser V. (2016). Electrochim. Acta.

[cit42] Ewert J.-K., Weingarth D., Denner C., Friedrich M., Zeiger M., Schreiber A., Jäckel N., Presser V., Kempe R. (2015). J. Mater. Chem. A.

[cit43] Yambou E. P., Beguin F. (2021). Electrochim. Acta.

[cit44] Gong Y., Li D., Luo C., Fu Q., Pan C. (2017). Green Chem..

[cit45] Li X., Tang Y., Song J., Yang W., Wang M., Zhu C., Zhao W., Zheng J., Lin Y. (2018). Carbon.

[cit46] Khomenko V., Raymundo-Pinero E., Frackowiak E., Beguin F. (2006). Appl. Phys. A: Mater. Sci. Process..

[cit47] Khan A., Senthil R. A., Pan J., Osman S., Sun Y., Shu X. (2020). Electrochim. Acta.

[cit48] Zhang Y., Shen Z., Yu Y., Liu L., Wang G., Chen A. (2018). J. Mater. Sci..

[cit49] Hassan M., Qiu W., Song X., Mao Q., Ren S., Hao C. (2020). J. Alloys Compd..

[cit50] Lee W.-h, Moon J. H. (2014). ACS Appl. Mater. Interfaces.

[cit51] Wang G., Wang R., Liu L., Zhang H., Du J., Zhang Y., Liu M., Liang K., Chen A. (2017). Mater. Lett..

[cit52] Sun G., Wang J., Li K., Li Y., Xie L. (2012). Electrochim. Acta.

[cit53] Du J., Yu Y., Liu L., Lv H., Chen A., Hou S. (2019). ACS Appl. Energy Mater..

[cit54] Bhattacharya S., Roy I., Tice A., Chapman C., Udangawa R., Chakrapani V., Plawsky J. L., Linhardt R. J. (2020). ACS Appl. Mater. Interfaces.

[cit55] Cui J., Yin J., Meng J., Liu Y., Liao M., Wu T., Dresselhaus M., Xie Y., Wu J., Lu C. (2021). Nano Lett..

[cit56] Ramu M., Chellan J. R., Goli N., Joaquim P., Cristobal V., Kim B. C. (2020). Adv. Funct. Mater..

[cit57] Kumar K. T., Sundari G. S., Kumar E. S., Ashwini A., Ramya M., Varsha P., Kalaivani R., Andikkadu M. S., Kumaran V., Gnanamuthu R. (2018). Mater. Lett..

