# Supplementary material for: Black goes green: single-step solvent exchange for sol-gel synthesis of carbon spherogels as high-performance supercapacitor electrodes
Source: Energy Adv. 2024 Jan 8;3(2):482–94. doi: 10.1039/d3ya00480e (PMC10867810; doi:10.1039/d3ya00480e)
Supplement: YA-003-D3YA00480E-s001 [file YA-003-D3YA00480E-s001.pdf]

Electronic Supplementary Material (ESI) for Energy Advances.
This journal is © The Royal Society of Chemistry 2024

Supporting Information

Black Goes Green: Single-Step Solvent Exchange for Sol-Gel Synthesis of Carbon Spherogels as High-Performance Supercapacitor Electrodes

Miralem Salihovic[a], Emmanuel Pameté[b], Stefanie Arnold[b,c], Irena Sulejmani[a], Theresa Bartschmid[a], Nicola Hüsing[a], Gerhard Fritz-Popovski[d], Chaochao Dun[e], Jeffrey J. Urban[e], Volker Presser[b,c,e*], and Michael S. Elsaesser[a*]

a *Chemistry and Physics of Materials, University of Salzburg, 5020 Salzburg, Austria*

b *INM – Leibniz Institute for New Materials, Campus D2 2, 66123, Saarbrücken, Germany*

c *Department of Materials Science & Engineering, Saarland University, Campus D2 2, 66123, Saarbrücken, Germany*

d *Institute of Physics, Montanuniversitaet Leoben, 8700 Leoben, Austria*

e *The MolecularFoundry, Lawrence Berkeley National Laboratory Berkeley, Berkeley, CA, 94720, USA*

f *Saarene – Saarland Center for Energy Materials and Sustainability, Campus C4 2, 66123 Saarbrücken, Germany*

* *Corresponding authors.* (VP) *volker.presser@leibniz-inm.de;* (ME) *michael.elsaesser@sbg.ac.at*

Supporting Information

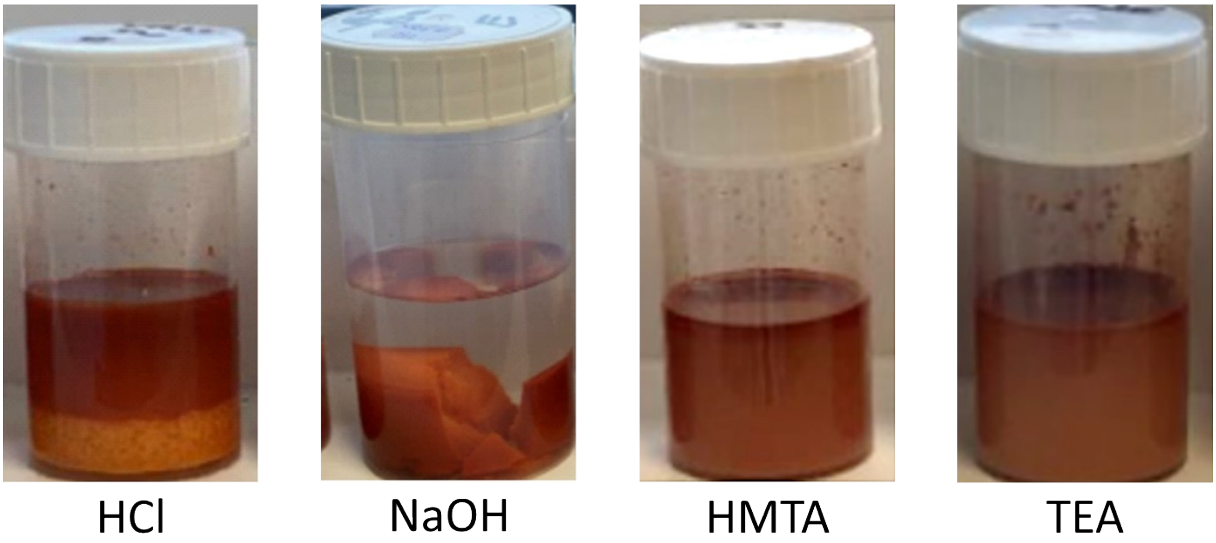

Figure S1: Photographs comparing the gel homogeneity of IPA-based RF/PS spherogels (R/IPA = 0.032, R/catalyst = 10, PS concentration in IPA = 2 mass%). The investigated catalysts from left to right: HCl, NaOH, HMTA, and TEA.

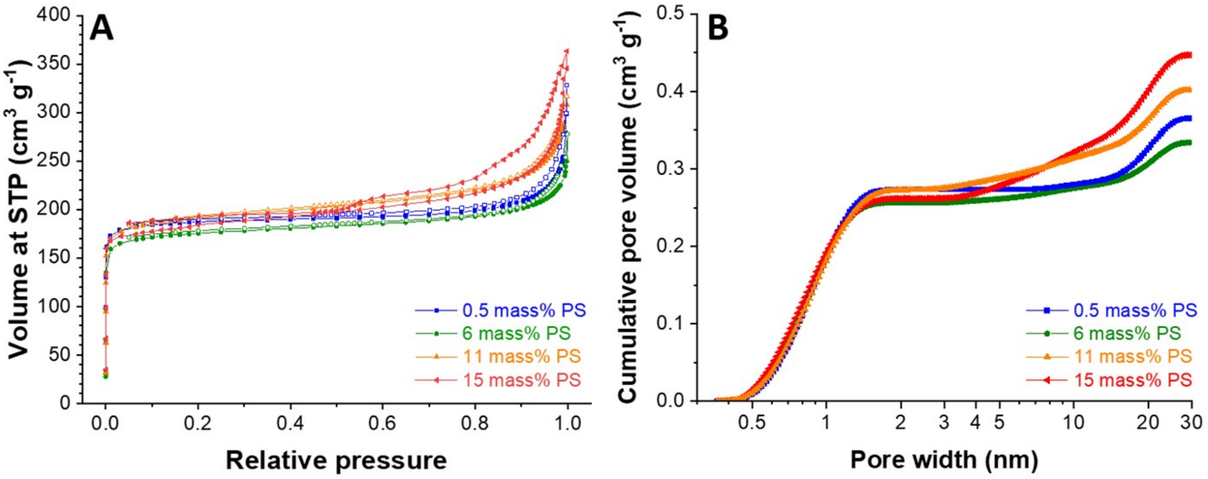

Figure S2: Nitrogen sorption isotherms of carbon spherogel samples derived from various polystyrene nanosphere concentrations in isopropyl alcohol (before sol preparation) (A). Isotherms were recorded at -196°C and in a relative pressure range p/p_0 from 10^{-7} to 1. Corresponding 2D-NLDFT cumulative pore size distribution pattern (B).

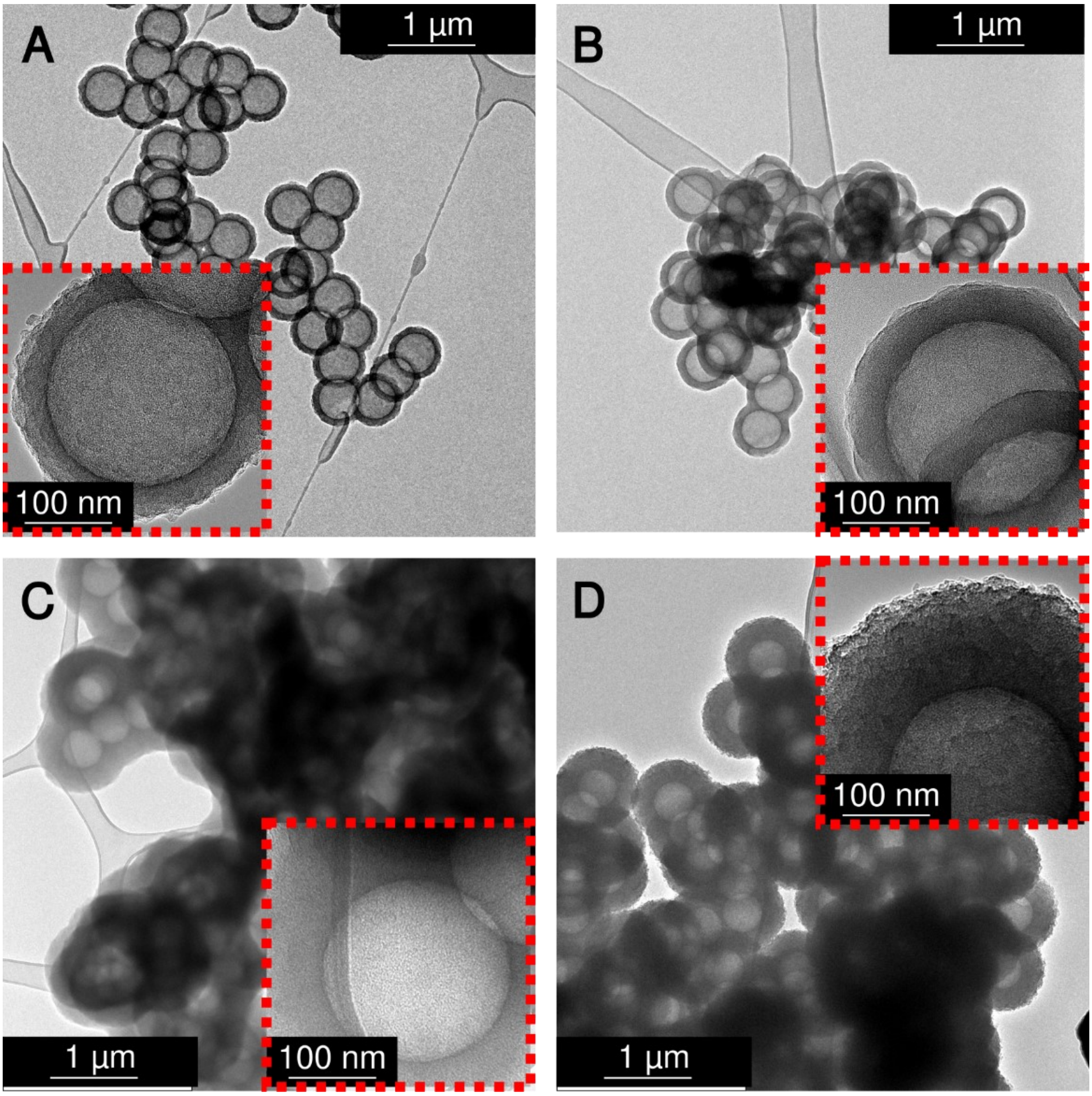

Figure S3: Transmission electron micrographs of directly carbonized (DC) and supercritically dried and carbonized (SCD) carbon spherogel samples **before** physical activation with CO_2. A: thin-walled sample (35 nm), SCD; B: thin-walled sample, DC; C: thick-walled sample (125 nm), SCD; D: thick-walled sample, DC.

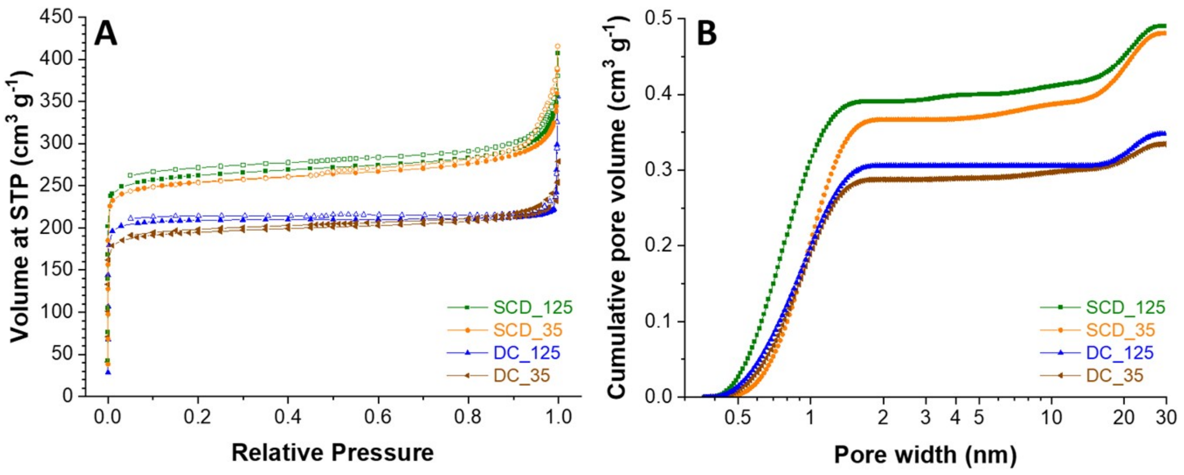

Figure S4: Nitrogen sorption isotherm comparison of directly carbonized (DC) and first supercritically dried and then carbonized (SCD) samples (non-activated) with thick and thin wall thickness: DC_125 and DC_35 as well as SCD_125 and SCD_35, respectively (A). Isotherms were recorded at -196°C and in a relative pressure range p/p_0 from 10^{-7} to 1. Corresponding cumulative pore size distributions evaluated by use of 2D-NLDFT (B).

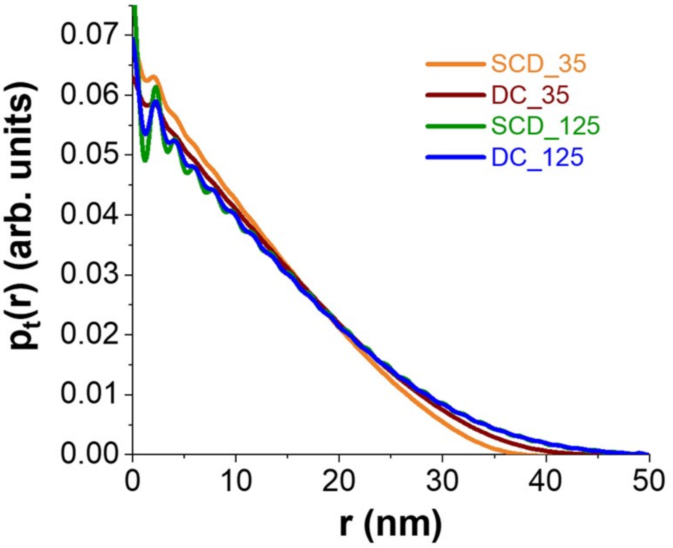

Figure S5: Thickness pair distance distribution function from indirect Fourier transformation.

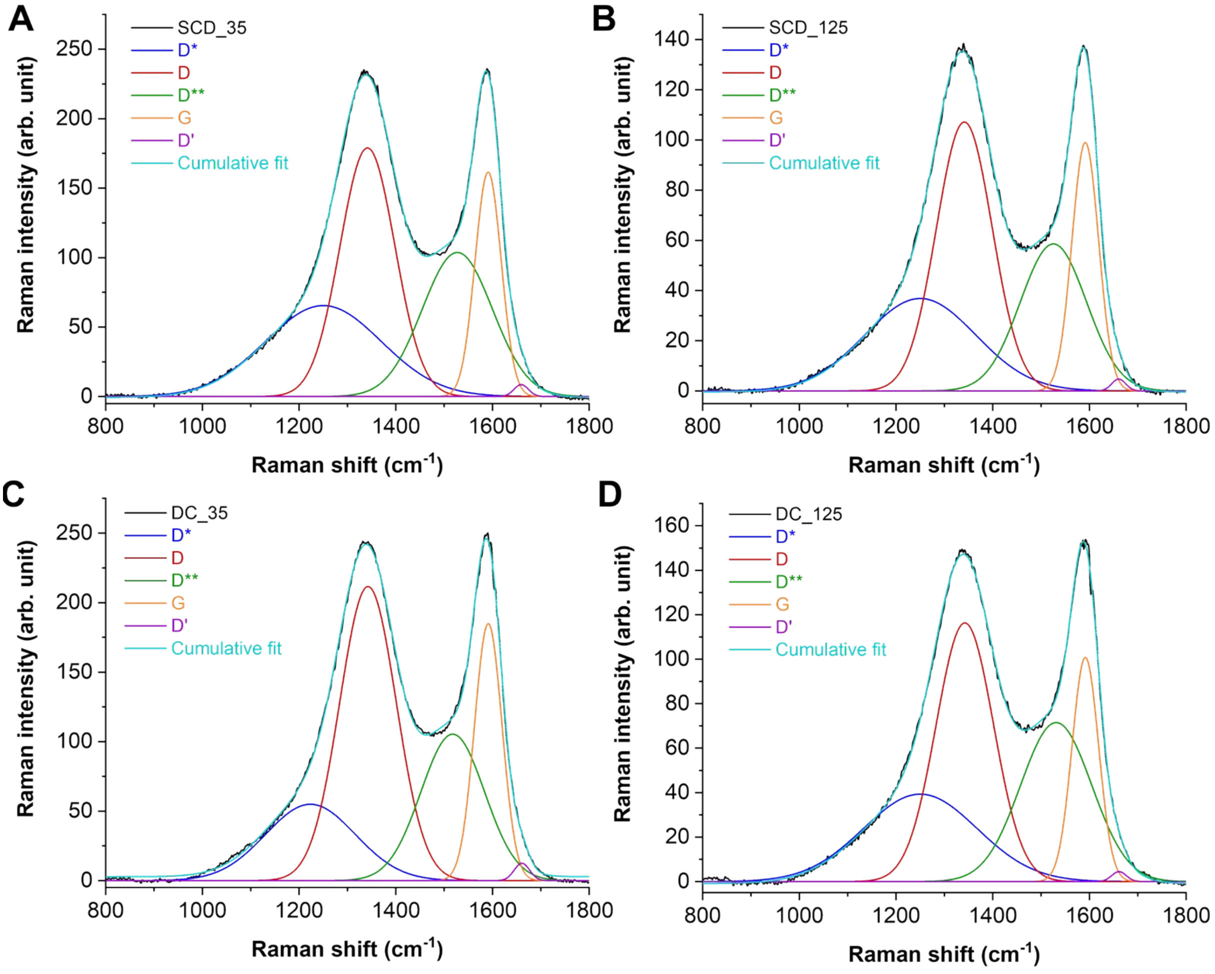

Figure S6: Raman spectra (black lines) and cumulative fit (light blue) of the carbon dioxide activated carbon spherogels, generated by supercritical drying with subsequent carbonization (A, B) and via direct carbonization (C, D) for 35 nm and 125 nm thick sphere walls, by deconvolution of the observed D- and G-bands into D* (blue), D (red), D** (green), G (yellow), and D’ (purple).

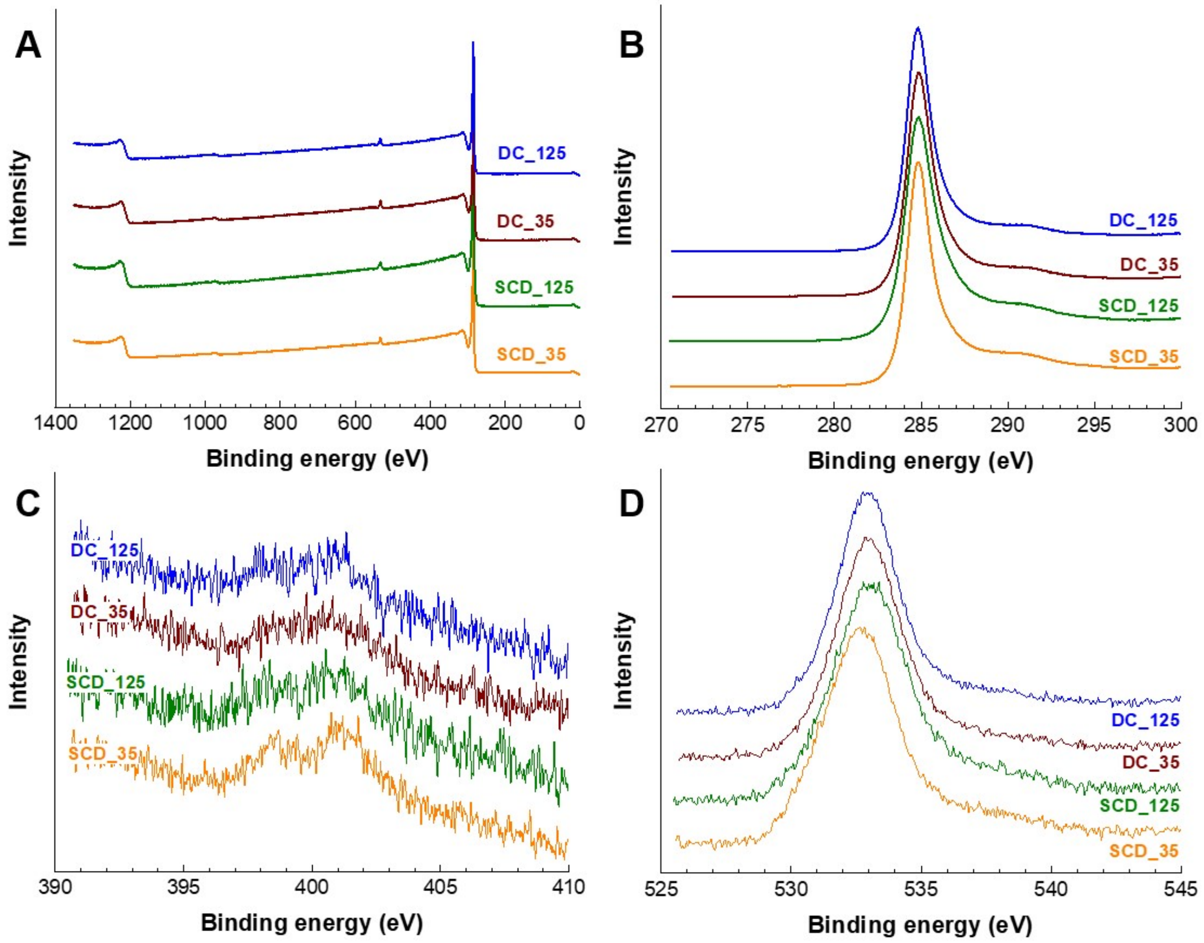

Figure S7: X-ray photoelectron spectra of IPA spherogel samples: (A) survey scan, (B) C 1s peak, (C) N peak, (D) O peak.

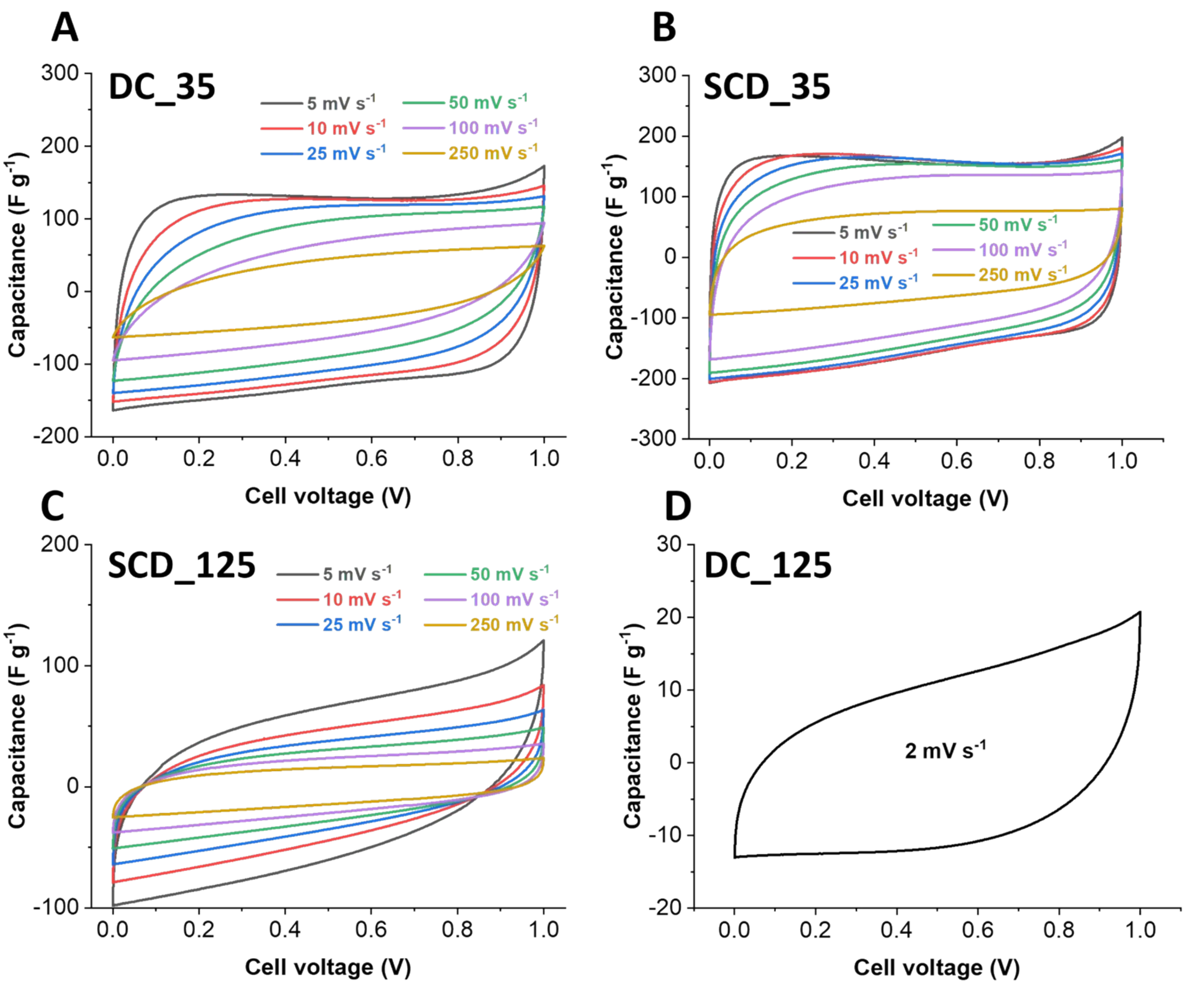

Figure S8: Electrochemical performance of different synthesized carbon spherogels. Cyclic voltammograms recorded at different scan rates of (A) DC_35, (B) SCD_35, (C) SCD_125, and (D) cyclic voltammogram at a scan rate of 2 mV s^{-1} from 0.0 V to 1.0 V of DC_125.

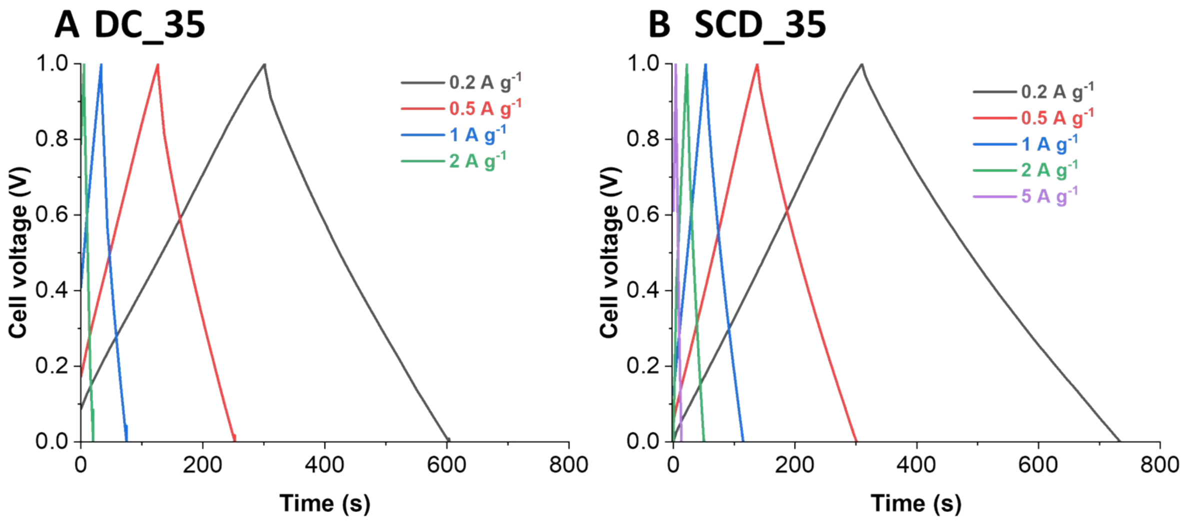

Figure S9: Electrochemical characterization showing the galvanostatic charge/discharge profiles of (A) DC_35 and (B) SCD_35 at different specific currents.

Table S1: Specific surface areas (SSA) and pore volume measurements relating to the wall thickness of carbon spherogels. Sample IDs correspond to **Figure 2** (n.a. = data not available).

**Sample ID**	**R/IPA**	**PS content (mass%)**	**Inner diameter* (nm)**	**Wall thickness* (nm)**	**SSA (2D-NLDFT) (m^2 g^{-1})**	**Pore volume at p p_0^{-1} = 0.98 (cm^3 g^{-1})**	**Micropore volume (cm^3 g^{-1})**
1	0.016	2	307±5	18±2	n.a.	n.a.	n.a.
2	0.032	2	301±14	57±2	n.a.	n.a.	n.a.
3	0.064	0.5	**	**	675	0.42	0.27
4	0.064	2	298±29	132±6	681	0.38	0.29
5	0.064	6	307±26	49±4	664	0.45	0.26
6	0.064	11	279±29	27±3	825	0.42	0.27
7	0.064	15	275±3	21±1	657	0.59	0.26
8	0.096	2	283±23	226±11	687	0.39	0.29
9	0.128	2	**	**	692	0.58	0.27

*average of 10 measurements (TEM)
**mixed structure: aerogel morphology containing separate hollow carbon spheres
